# Supplementary material for: PTSD symptoms among health workers and public service providers during the COVID-19 outbreak
Source: PLoS One. 2020 Oct 21;15(10):e0241032. doi: 10.1371/journal.pone.0241032 (PMC7577493; doi:10.1371/journal.pone.0241032)
Supplement: S1 File — (DOCX) [file pone.0241032.s001.docx]

**S1 File**

**Overview of questionnaires and items**

**PTSD Checklist for DSM-5 (PCL-5)**

Below is a list of problems that people sometimes have in response to a very stressful experience. Please read each problem carefully and then circle one of the numbers to the right to indicate how much you have been bothered by that problem in the past month.

In the past month, how much were you bothered by:

1. Repeated, disturbing, and unwanted memories of the stressful experience?
2. Repeated, disturbing dreams of the stressful experience?
3. Suddenly feeling or acting as if the stressful experience were actually happening again (as if you were actually back there reliving it)?
4. Feeling very upset when something reminded you of the stressful experience?
5. Having strong physical reactions when something reminded you of the stressful experience (for example, heart pounding, trouble breathing, sweating)?
6. Avoiding memories, thoughts, or feelings related to the stressful experience?
7. Avoiding external reminders of the stressful experience (for example, people, places, conversations, activities, objects, or situations)?
8. Trouble remembering important parts of the stressful experience?
9. Having strong negative beliefs about yourself, other people, or the world (for example, having thoughts such as: I am bad, there is something seriously wrong with me, no one can be trusted, the world is completely dangerous)?
10. Blaming yourself or someone else for the stressful experience or what happened after it?
11. Having strong negative feelings such as fear, horror, anger, guilt, or shame?
12. Loss of interest in activities that you used to enjoy?
13. Feeling distant or cut off from other people?
14. Trouble experiencing positive feelings (for example, being unable to feel happiness or have loving feelings for people close to you)?
15. Irritable behavior, angry outbursts, or acting aggressively?
16. Taking too many risks or doing things that could cause you harm?
17. Being “superalert” or watchful or on guard?
18. Feeling jumpy or easily startled?
19. Having difficulty concentrating?
20. Trouble falling or staying asleep?

**Cognitive Attentional Syndrome Scale (CAS-1)**

Below are a number of beliefs people have. Indicate how much you believe each one by placing a number from the scale below next to each item:

Negative metacognitions:

1. Worrying too much could harm me.
2. Strong emotions are dangerous
3. I cannot control my thoughts
4. Some thoughts could make me lose my mind.

Positive metacognitions

1. Worrying helps me cope.
2. Focusing on possible threat can keep me safe.
3. It is important to control my thoughts.
4. Analyzing my problems will help me find answers.

**Selected items from Inventory of Interpersonal Problems (IIP-64):**

1. I find it difficult to assert my own opinions towards another person.
2. I find it difficult to confront people with problems that arise.
3. I find it difficult to express my feelings to others directly.
4. I do what is best for me when another person becomes demanding.
5. I often let other people’s needs take precedence over my own.
6. I find it difficult to argue with another person
7. I find it difficult to show other people that I care about them.
8. I find it difficult to feel close to others.
9. I find it difficult to open up to talk about my feelings to others.
10. I desire to be noticed way too often.
11. I often speak my mind without worrying about hurting someone else's feelings.
12. I find it difficult to spend time alone
13. It is hard for me to ask another person to stop bothering me
14. It is hard for me to set boundaries for others
15. I find it difficult to forgive another person after being angry
16. I find it difficult to understand the perspective of others
17. I try to change others too often

**Health Anxiety**

Over the last 2 weeks, how often have did the following statements apply to you?

**Selected items from Health Anxiety Inventory (HAI):**

1. I spend most of my time worrying about my health
2. I constantly have images of myself being ill.
3. I worry about being infected by the coronavirus.
4. I fear dying from the coronavirus.

**Emotional support:**

Over the last 2 weeks, how often have did the following statements apply to you?

1. I feel that I receive sufficient social support from others.
2. I feel others close to me care about me.
3. I feel close and connected with other people who are important to me.

**Worry about job and economy:**

Over the last 2 weeks, how often have did the following statements apply to you?

1. I am worried about losing my job.
2. I am worried about my economy.

**Burnout**

Over the last 2 weeks, how often did the following statement apply to you?

1. I feel burned out.

**Demographic questions**

1. What is your biological sex?
2. Is your self-identified sex the same as your biologically assigned sex?
3. How old are you?
4. What is your civil status?
5. What is your educational level?
6. Are you currently living with any of your children?
7. Indicate whether you have a pre-existing mental health condition as revealed by a psychiatric diagnosis.

**Vulnerable professional occupation:**

1. What is your profession?
   1. Doctor
   2. Psychologist
   3. Nurse
   4. Social worker
   5. Politician working with pandemic protocols and pandemic consequences
   6. Other health-care professionals
   7. Other

**Direct vs. indirect**

1. Do you work directly or indirectly with the COVID-19 virus?

- 1. Directly is defined as having face-to-face contact with a patient infected the COVID-virus.
  2. Indirectly involves working with individuals with change life circumstances due to the virus or working with the pandemic consequences on state-level, without direct exposure to patients infected by the virus.

**Patient Health Questionnaire (PHQ-9)**

Over the last 2 weeks, how often have you been bothered by any of the following problems?

1. Little interest or pleasure in doing things
2. Feeling down, depressed, or hopeless
3. Trouble falling or staying asleep, or sleeping too much
4. Feeling tired or having little energy
5. Poor appetite or overeating
6. Feeling bad about yourself or that you are a failure or have let yourself or your family down
7. Trouble concentrating on things, such as reading the newspaper or watching television
8. Moving or speaking so slowly that other people could have noticed. Or the opposite being so figety or restless that you have been moving around a lot more than usual
9. Thoughts that you would be better off dead, or of hurting yourself

**Generalized Anxiety Disorder 7 (GAD-7)**

Over the last 2 weeks, how often have you been bothered by the following problems?

1. Feeling nervous, anxious, or on edge
2. Not being able to stop or control worrying
3. Worrying too much about different things
4. Trouble relaxing
5. Being so restless that it's hard to sit still
6. Becoming easily annoyed or irritable
7. Feeling afraid as if something awful might happen
