# Supplementary material for: PTSD symptoms among health workers and public service providers during the COVID-19 outbreak
Source: PLoS One. 2020 Oct 21;15(10):e0241032. doi: 10.1371/journal.pone.0241032 (PMC7577493; doi:10.1371/journal.pone.0241032)
Supplement: Attachment — Submitted filename: Response to Reviewers.docx [file pone.0241032.s002.docx]

**Response to Reviewers**

PONE-D-20-19419
PTSD-symptoms Among Health Workers and Public Service Providers During the COVID-19 Outbreak

We would like to thank the reviewers and editorial team for their comments on the paper, which gave us the opportunity to make improvements in the manuscript. Detailed responses to reviewers’ comments are given for each point separately. Each response is identified by the number of the reviewers and the number of the comment. Our responses are given in italics.

1. Please ensure that your manuscript meets PLOS ONE's style requirements, including those for file naming. The PLOS ONE style templates can be found at

https://journals.plos.org/plosone/s/file?id=wjVg/PLOSOne_formatting_sample_main_body.pdf and

<https://journals.plos.org/plosone/s/file?id=ba62/PLOSOne_formatting_sample_title_authors_affiliations.pdf>

*The PLOS ONE style requirements have been revisited, and the paper should currently meet these requirements.*

2. We note that you have indicated that data from this study are available upon request. PLOS only allows data to be available upon request if there are legal or ethical restrictions on sharing data publicly. For information on unacceptable data access restrictions, please see http://journals.plos.org/plosone/s/data-availability#loc-unacceptable-data-access-restrictions.

In your revised cover letter, please address the following prompts:

a) If there are ethical or legal restrictions on sharing a de-identified data set, please explain them in detail (e.g., data contain potentially identifying or sensitive patient information) and who has imposed them (e.g., an ethics committee). Please also provide contact information for a data access committee, ethics committee, or other institutional body to which data requests may be sent.

b) If there are no restrictions, please upload the minimal anonymized data set necessary to replicate your study findings as either Supporting Information files or to a stable, public repository and provide us with the relevant URLs, DOIs, or accession numbers. Please see http://www.bmj.com/content/340/bmj.c181.long for guidelines on how to de-identify and prepare clinical data for publication. For a list of acceptable repositories, please see http://journals.plos.org/plosone/s/data-availability#loc-recommended-repositories.

We will update your Data Availability statement on your behalf to reflect the information you provide.

*As requested by the editor I have been in contact with NSD and REK.*

*The present dataset includes information about specific behaviors, trait variables, and health outcomes of a large and representative sample of the adults. In accordance with the guidelines of The Centre for Research Data (NSD) and The Regional Committee for Medical and Health Research Ethics (REK), access to the data can only be provided to qualified investigators whose proposed use of the data have been approved by all relevant independent review committees in the conducted countries of the study, including REK and NSD, and whose research plans provide a defensible proposal and methodologically sound design with the aims approved by REK, NSD, the university ethics board, and other necessary organizations that require an application process for providing access to sensitive data containing predictive information on a large and representative sample of the adult population in the sample country.*

*The main reasons for the constrain on the use of the data is the following. First, free use of the data, even in anonymized form, is not mentioned in the letter of informed consent, which is a prerequisite for the study. Second, the deposit of anonymized data, on an international server is not allowed according the ethical board based on our informed consent. However as specified, the data can be requested from the first author following ethical approval of access to the data from NSD and REK. REK and NSD does not handle request for the use of data on behalf of the author.*

*We suggest the following data availability statement which is in accordance with other papers from Norway published in PLOS ONE:*

*Suggestion for Data Availability Statement:*

*Our ethical approval granted by the Regional Committees for Medical and Health Research Ethics in Norway does not allow us to submit the data to a Public repository. In line with the ethics approval, the data are to be kept at a secure server only accessible by the authors at the University of Oslo. Access to the data can be granted from the first author following ethical approval of suggested project plan for the use of data from NSD and REK. Such requests are to be sent to Associate Professor, Sverre Urnes Johnson, Department of Psychology, University of Oslo, Forskningsveien 3 A, 0373 Oslo, Norway, Email: s.u.johnson@psykologi.uio.no, phone: +47-22845295*

3. Please upload a copy of the Supporting Information which you refer to in your text on page

*A copy of the supplementary material encompassing all the different items used in the study is now uploaded.*

Reviewer #1:

The authors present findings from a large cohort size, reporting the prevalence of PTSD symptoms amongst their cohort. This manuscript may not be novel, but it does add to the body of literature to help understand the psychological impact of covid both in Norway and Globally. The overall methodology and analyses is robust. A few minor amendments is suggested.

1. In discussion, please attempt to compare PTSD symptom prevalence to other countries and discuss accordingly: e.g. Chew NW, Lee GK, et al. A multinational, multicentre study on the psychological outcomes and associated physical symptoms amongst healthcare workers during COVID-19 outbreak. Brain, behavior, and immunity. 2020 Apr 21.

*The paper by Chew et al is included in the revised manuscript.*

*The results from the current paper is discussed in comparison with other studies, including the study from Chew et al: See page 12: “However in a recent study, PTSD symptoms among health-workers in India and Singapore during the COVID-19 outbreak the prevalence-rate of 9.3% experiencing PTSD-symptoms, were lower than in the current study [43]. There could be several reasons for differences in prevalence-rates between countries and firm conclusions are hampered by the use of different measures to assess PTSD symptoms. It is suggested, however, that that some western countries have higher risk of PTSD because there are high expectations for risk-fee life and high attention to potential harmful mental health effects of serious life events [44]”.*

-Although other papers may use different instruments to measure PTSD and this is an inherent limitation.

*This is a clear limitation, and is mentioned in the discussion, see page 12.*

--did factors like case volume in the country, healthcare resources strain, mortality rate play a role?

*Thank you for a good suggestion. Norway did not have extraordinary many deaths related to Covid-19 and normally you would expect that more strain on healthcare resources and higher mortality rate would be associated with higher PTSD-symptoms, but this does not seem to be the case. India have for example higher mortality rate compared to Norway (*[*https://coronavirus.jhu.edu/data/mortality*](https://coronavirus.jhu.edu/data/mortality)*). Thus, the paper by Heir et al about possible higher prevalence of PTSD in the certain western populations is relevant and mentioned in the discussion (page 12).*

2. one major limitation is that it is a cross-sectional study. we are unsure how much of this is related to covid directly, or may have been pre-existing prior to covid. the authors should include this as a limitation. further longitudinal study in a similar cohort can also be used to assess the long-term PTSD impact once the pandemic blows over.

*We have highlighted that the cross-sectional methodology hinders causal conclusion, see page 15. Further longitudinal studies of the same cohort are planned.*

Reviewer #2: The authors present a timely examination of PTSD, depression, and anxiety in relation to COVID-19 pandemic in a large sample of healthcare providers out of Norway. While this is a large study with self-report data, the timeliness of the topic combined with efficient and well-done study design (in choice of measures, etc.) make this manuscript a useful contribution to the literature as we try to figure out what needs to be done to reduce the mental health impacts of the current pandemic.
Introduction is concise and well written to support the design.

Methods are good and results clearly presented.

Discussion is well reasoned, sticks to the results presented and provides a key contribution to the literature.

*Thank you for the positive feedback.*

Given the journal requirements to provide data or a rationale why data cannot be provided, I believe additional rationale beyond ethics needs to be provided, but that is my only hesitation.

*The reason for unavailability of sharing through a deposit of anonymized data is given in the beginning of this document.*

Reviewer #3: Thank you for the opportunity to review this manuscript. The authors conducted a timely examination of self-reported symptoms for front-line workers during the current global pandemic. The novelty of examining public service workers is unique to the study, but not as distinguished in both the methodology nor discussion. Rather, the study methodologies, in particular, the recruitment of target populations was not supported by, or in line with the strong theoretical rationale. Below, I outline some of my thoughts.


Background and introduction:
1. Background is well-written, with strong rationale in conducting this research.

*Thank you*

Methods:
1. Some clarifications are needed as there seems to be a disconnect between the background rationale and study methodology. While the examination of healthcare and public service providers are clear from the background and rationale, the exact recruitment and populations reached are not. For example, which groups were considered healthcare professional – those working in ER/COVID units? Nurses in long-term homes? What about other healthcare professionals, and particularly for elective procedures, like mental health professionals, family physician, physiotherapists, pharmacists, etc.

*We have created at new paragraph, where we explain in further details the recruitment procedure. “The participants were either health personnel or public service providers working directly or indirectly with COVID-19 patients. The following groups of health personnel were assessed: Medical doctors, nurses, clinical psychologist and other health workers (not specified). The following group of public service providers working with consequences of the COVID-19 pandemic were assessed and grouped: social workers, politicians and other professions (not specified).”*

*As specified we did not assess the level of detail as the reviewer suggest, thus a category of “other” exist both for health personnel and public service providers.*

2. Similarly, for public service professionals, what is considered "public service"? What about essential workers such as bus drivers, grocery store clerks, who likely have less protection compared to healthcare professionals, earn a lower wage, and are still required to work during the pandemic? Are they factored into your recruitment strategy?

*Public service is a broad term, but in the questionnaire the work had to be related to consequences of the pandemic and those answering as public service providers worked indirectly with the consequences. However, the questionnaire was not detailed enough to separate between grocery store clerks, bus drivers etc. We have specified the term public service provider, see page 7: “The following group of public service providers working with consequences of the COVID-19 pandemic were assessed and grouped: social workers, politicians and other professions (not specified)”.*

3. The recruitment strategy is missing some details. For example, what did the Facebook algorithm determine for health personnel and public service providers, and who were the politicians reached?

The following information is given on page 6:

*“The Facebook algorithm reaches a random sample of individuals including and above 18 years of age who have reported their labor to fit our target categories, such as nurses, doctors, psychologists, and other individuals working in the health-care system, in addition to those reported to be politicians and social workers. The algorithm is inherently designed to optimize for genuine human activity and uses a variety of methods to remove false and duplicate accounts from the selection algorithm. Upon taking the survey, these participants identify and register themselves through a platform referred to as Services for Sensitive Data (TSD), where their information is safely stored. With the imputed parameters, the algorithm reached a total of 12 113 individuals meeting the aforementioned criteria.”.*

4. For public service providers, there was only options for social workers and politicians. This is not reflective of the diversity of public service providers nor exhaustive enough to draw conclusions from.

*This is a relevant point, and the lack of diversity of the construct “public service providers” is now mentioned as a limitation at page 15: “The group public service providers had lack of diversity consisting of only social workers and politicians reducing the possibility to generalize across this group.”*

5. Demographic variables did not consider contact and exposure to covid-19, which may be central to this line of research.

*We did have questions about contact and exposure to Covid-19 and whether the person was in quarantine, but we did not use the variables in this paper, because we think the variables are less central for the questions asked in this study and the variables were used in another publication:*

*Ebrahimi OV, Hoffart A, Johnson SU. The mental health impact of non-pharmacological interventions aimed at impeding viral transmission during the COVID-19 pandemic in a general adult population and the factors associated with adherence to these mitigation strategies. 2020, May 9. doi:* [*https://doi.org/10.31234/osf.io/kjzsp*](https://doi.org/10.31234/osf.io/kjzsp)*.*

6. Please clarify experimental groupings and rationale. Which groups/professions were considered healthcare workers vs. public service workers. These were not mentioned until demographic characteristics in the results section.

*The groups are clarified and mentioned below the heading “participants”, see page 7:*

*“The participants were either health personnel or public service providers working directly or indirectly with COVID-19 patients. The following groups of health personnel were assessed: Medical doctors, nurses, clinical psychologist and other health workers (not specified). The following group of public service providers working with consequences of the COVID-19 pandemic were assessed and grouped: social workers, politicians and other professions (not specified).”*

7. I was not able to locate the supplementary file for the single item measure of burnout. How was this construct quantified/operationalized? If it’s a single item, please insert item right into text.

*A supplementary file consisting of all the questionnaires used in the study is uploaded. Furthermore, the single items “I feel burned out” is inserted in the text.*

Results:
1. Unclear about direct vs indirect work relationships with COVID-19 from both methodology and results. How was this operationalized, was frequency of contact, size of hospital/healthcare institution, presence of outbreaks, and other variables considered?

*As specified on page 5 at the end of the introduction:*

*Directly is defined as face-to-face contact with patients that have tested positive for COVID-19. Indirectly is defined as working with other consequences of the COVID-19 pandemic, but not face-to-face with patients that is infected with COVID-19.*

2. Please fix "other predictors of treatment outcome". This is a cross-sectional inferential analysis, no treatment was offered.

*The sentence is changed to “Other predictors of PTSD symptoms”.*

Discussion:
1. Given the focus of the discussion on distinctions between public service vs. healthcare professionals, it is important to clearly operationalize and provide clear details about recruitment strategies and conditions for inclusion/exclusion.

*We have tried to make the information about the two groups clearer throughout the manuscript.*

2. With small proportion of public service professionals sampled, and lack of clarify regarding the types and diversity of profession, I caution the authors in drawing such strong conclusions from data collected. Would recommend to highlight as a limitation in data interpretations.

*We have highlighted the heterogeneity of the group “public service providers” as a limitation, see page 15: “The group public service providers had lack of diversity consisting of only social workers and politicians reducing the possibility to generalize across this group.”*
