# Supplementary material for: PTSD symptoms among health workers and public service providers during the COVID-19 outbreak
Source: PLoS One. 2020 Oct 21;15(10):e0241032. doi: 10.1371/journal.pone.0241032 (PMC7577493; doi:10.1371/journal.pone.0241032)
Supplement: Attachment — Submitted filename: Response to Reviewers 5.10.2020.docx [file pone.0241032.s003.docx]

Revision letter 5.10.2020

PONE-D-20-19419
PTSD-symptoms Among Health Workers and Public Service Providers During the COVID-19 Outbreak

We would like to thank the reviewers and editorial team for their comments on related manuscripts. Detailed responses to editor comments (E) are given for each point separately, behind the letter A.

E1: PLOS ONE has guidelines when different publications are related. If there is no overlap between this manuscript and the other publication (Ebrahimi et al.), please let me know that. If there is any overlap, please follow the PLOS ONE guidelines at
<https://journals.plos.org/plosone/s/submission-guidelines#loc-related-manuscripts>

A: It is unclear for the authors how PLOS ONE defines “overlap”, but for the sake of transparency we have given a statement about any potential overlap.

E2: Specifically, upon your submission of your revised manuscript, please indicate whether there are any related manuscripts under consideration or published elsewhere. If related work has been submitted or published elsewhere, please include a copy of it with your revised manuscript and describe its relation to the submitted work.

A2: We have included a copy of three separate papers from the same dataset which have been submitted for publication. We don`t think that there are any substantial overlap since the first, Ebrahimi et al, targets the general population for anxiety and depression, Hoffart et al., targets loneliness in the general population and Johnson et al., targets parental stress in the general population. However, the data from the same data-collection.

E3: I will also ask that that the authors make it clear in the revised manuscript that results from a related dataset have been published previously or are under consideration for publication, if applicable.

A3: In the revised manuscript the following information is added: “Articles from the same project, but with different topics, concerning the prevalence of anxiety and depression [30], loneliness [31]and parental stress [32] are under consideration for publication.”
